# Abdominal Ultrasonography in Healthy Female Standard Donkeys

**DOI:** 10.3390/ani15020129

**Published:** 2025-01-08

**Authors:** Lucrezia Accorroni, Marilena Bazzano, Andrea Marchegiani, Andrea Spaterna, Fulvio Laus

**Affiliations:** School of Biosciences and Veterinary Medicine, University of Camerino, Via Circonvallazione, 93/95, 62024 Matelica, Italyfulvio.laus@unicam.it (F.L.)

**Keywords:** ultrasonography, abdomen, donkey

## Abstract

Knowledge of the normal ultrasound anatomy in the donkey is essential to understand pathological changes and to reach an early diagnosis, considering the “dullness” and stoicism of these animals in pain manifestations. The aims of this study were to document which abdominal viscera could be identified using a transcutaneous ultrasonography technique under field conditions in unsedated, standing donkeys to determine where the different abdominal structures could be easily displayed and to describe any variation that occurred between different donkeys and horses. In this work, the manual technique for performing an ultrasound of the abdominal organs in a donkey is described, including acoustic windows. In addition, several measurements were performed and correlated with the age and weight of the animal to find possible variations. For several of the abdominal viscera, donkeys were found to have similarities with horses, but some differences were found, mostly related to kidney size and the technique for liver visualization. The wall thickness of the colon was confirmed as higher compared to horses, while other intestinal tracts were found to be comparable. A correlation between body weight and organ measurements was only found for the length of both kidneys.

## Simple Summary

Transabdominal ultrasonographic scanning of a donkey’s abdomen is an important diagnostic tool that can be used to assess the health of the animal. The aims of this study were to validate the use of transcutaneous ultrasonography under field conditions in donkeys to document which abdominal viscera could be easily identified and where the best acoustic windows are located and to describe any variation that occurred between different donkeys and horses. An ultrasonographic examination of the abdomen was found to be safely and easily performed in donkeys under field conditions and without sedation. The findings of the present study are in some instances compatible with those reported in horses for spleen, stomach, duodenum, colon and cecum; however, there are differences for the liver extension and kidney size and location. The recorded mean and standard deviations for the wall thickness of the different gastrointestinal tracts in the examined donkeys were inside the range reported for donkeys and horses, except for the colon wall. Due to the low tendency of donkeys to show clinical symptoms of pain or discomfort compared to horses, ultrasound acquires considerable importance as an early diagnostic tool in the evaluation of this species.

## 1. Introduction

Abdominal ultrasonography (US) is regularly performed in the clinical practice of equids [1] as a complementary tool in the decision-making workup for horses with acute visceral signs and is useful when rectal palpation is impossible or unacceptably risky for both horse and veterinarian [2,3].

Rectal palpation allows for the evaluation of only 25–35% of the peritoneal cavity in cases of suspected intra-abdominal disease, and in small breeds and foals, it cannot be easily performed due to the animal’s size and their temperament, which sometimes place the horse and the veterinarian at risk of injury [4,5].

Despite the large size of the abdominal cavity, which prevents the complete examination of the digestive tract and abdominal organs in equine patients [4], US (ultrasound) allows for the visualization of anatomical structures that are inaccessible by rectal palpation, giving complementary data that are useful for evaluating the clinical case and representing a useful additional diagnostic tool [6]. Donkeys generally have a milder temperament than horses, but their size is usually smaller, making a rectal examination difficult or impossible to be performed and US a suitable and appealing complementary test to perform [7].

The knowledge of normal ultrasound abdominal anatomy and image optimization in clinically healthy horses is well known, but for donkeys, this information, which is fundamental to obtain diagnostic images and to understand pathologic changes in case of disease, is not completely ascertained [8,9].

Transcutaneous abdominal ultrasonography is a fast and non-invasive tool that gives immediate information about the location, volume and pattern of abdominal viscera; the presence and type of peritoneal fluid; the intestinal motility; the wall thickness; and the luminal contents [5,8]. Donkeys have been recently rediscovered for milk production as an alternative food source for milk-intolerant children [10,11], and their current popularity is also due to their use as pets, in addition to recreational purposes, sports activities, donkey-assisted therapy and, to a lesser extent, as pack/draught animals and for meat production [4,12]. Given their importance in society and their growing numbers, the improvement of veterinary management and diagnostic workup must occur accordingly [13].

The abdominal scanning technique is well known and standardized in horses, especially for the identification of gastrointestinal structures and anomalies in cases of colic [1,14]. On the contrary, only one study has been carried out on this topic in donkeys, which was limited to the gastrointestinal tract, and a real description of the operating procedure is not yet available [8]. Furthermore, there are a limited number of studies comparing donkeys’ and horses’ ultrasonographic anatomies [15,16].

The aim of this study was to evaluate the feasibility of the transcutaneous ultrasonography technique under field conditions in unsedated standing donkeys and to detect which different abdominal structures can be displayed.

## 2. Materials and Methods

### 2.1. Donkeys

Seventeen adult female standard donkeys (*Equus asinus*) were enrolled in this study. Their ages ranged between 2 and 17 years (average: 10 ± 5 years) and their weights between 113 and 326 kg (average 187 ± 87 kg). A complete physical examination and clinical abdominal evaluation were performed before the US scan, and only clinically healthy and not pregnant donkeys were included in the study. All animals were selected to be easily manageable to avoid sedation. The faeces of all animals were macroscopically observed to detect any alterations in consistency, colour and odour. The faeces were also investigated for the presence of parasites by microscopic evaluation, and only negative donkeys were included in the study. The US examinations were performed between March and April 2024; all jennies were kept indoor in the same stable with access to an external paddock and underwent the same management practices; they were fed with hay at the same time, which was two hours before the examination.

All procedures were compliant with Directive 2010/63/EU on the protection of animals used for scientific purposes.

### 2.2. Ultrasonographic Examination

Ultrasound examinations were performed in an open but covered environment in order to have only soft light that would not compromise the visualization and evaluation of the images. The US was carried out using a Sonosite M-Turbo scanner (FUJIFILM Sonosite Europe, Joope Geesinkweg, 140, Amsterdam, The Netherlands) with a C60xi multifrequency (2–5 MHz) convex transducer, used at 5 MHz. Acoustic windows were brushed and then soaked with 90° isopropyl alcohol to remove fat particles and to ensure the best possible contact between the transducer and the skin.

The US exam started in the left and right paralumbar fossa and was carried out cranially until the abdominal organs were no longer visible. The transducer was moved in a dorsal to ventral direction. Furthermore, the probe was placed ventrally in the proximal third of the neck for oesophagus visualization.

The location and wall thickness of the oesophagus, stomach, duodenum, left colon, right colon and cecum were assessed. Moreover, images of the kidney, liver and spleen were obtained. Each measurement was taken three times by the same operator, and the mean value was used for statistical analysis.

### 2.3. Statistical Analysis

GraphPad Prism version 8.2.1 for macOS (GraphPad Software, La Jolla, San Diego, CA, USA) was used to perform the statistical analyses.

Descriptive statistics were reported (mean (SD), 95% IC, Percentile, Min, Max and Range). First, the data were checked using the Shapiro–Wilk test for normality. The relationships between age and body weight with all measurements were assessed by the Pearson correlation coefficient. A value of *p* < 0.05 was considered significant.

## 3. Results

**Figure 1:**
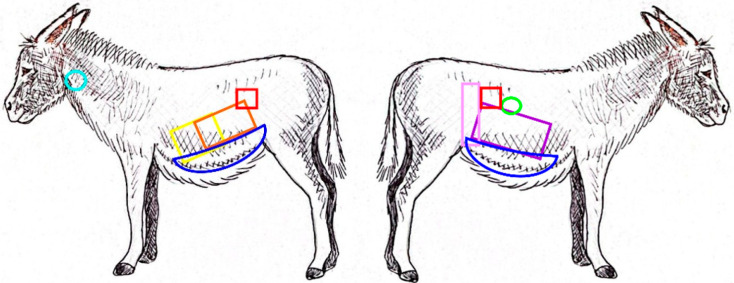
Acoustic windows of the left and right side of the abdomen, respectively. Blue: colon; red: kidney; light blue: oesophagus; yellow: stomach; orange: spleen; pink: caecum; purple: liver; green: duodenum.

All the examinations were easily performed without the need for sedation, and all patients were cooperative, without displaying any sign of discomfort or stress during the procedures. Figure 1 reports the acoustic windows that were used to visualize the abdominal viscera, whose measurements are reported in Table 1 and Table 2.

**Figure 2:**
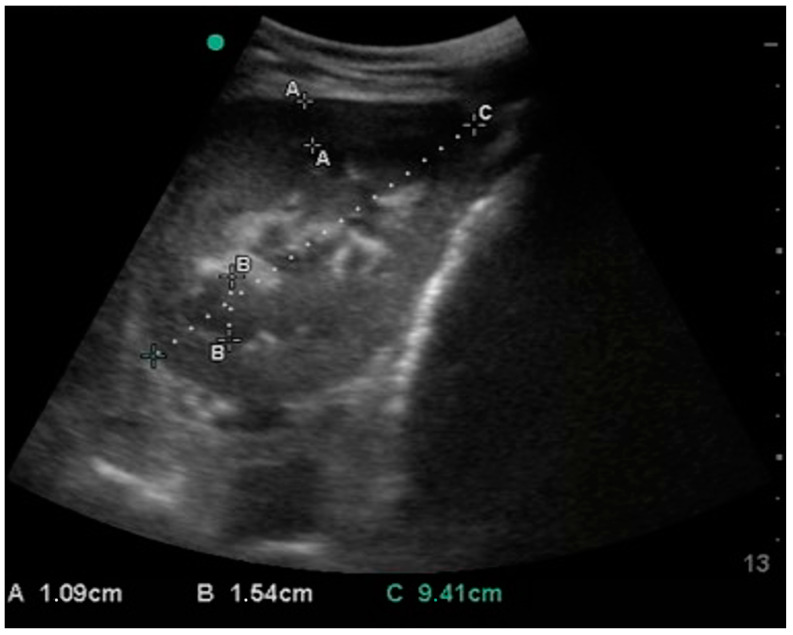
Sonogram of the right kidney, obtained from the 17th ICS. Cortex (**A**), medulla (**B**) and length (**C**) measurements are reported. The green dot represents the marker of the probe; the left side of this image is dorsal, and the right side is ventral.

The left kidney was visualized on the paralumbar fossa at the level of the tuber coxae in all the donkeys. The right kidney (Figure 2) was scanned in the 17th intercostal space (ICS) in 12 cases (71%), in the paralumbar fossa in 4 (23%) and in the 16th in 1 case (6%).

The liver was easily displayed from the 11th to the 15th ICS in all the donkeys, but in some cases (24%), it could extend for two additional ICSs caudally and/or cranially. Hepatic vessels and bile ducts were diffusely seen in all the parenchyma.

The spleen was visible in all animals from the 12th to the 15th ICS. As for the liver, the spleen was displayed up to the 8th ICS cranially and to the paralumbar fossa caudally in the donkeys included in the study. The splenic vein, in the medial aspect of the organ, was located caudally and dorsally to the stomach. As for the horse and other domestic animals, the spleen was found to be more echogenic and homogeneous than the liver.

**Figure 3:**
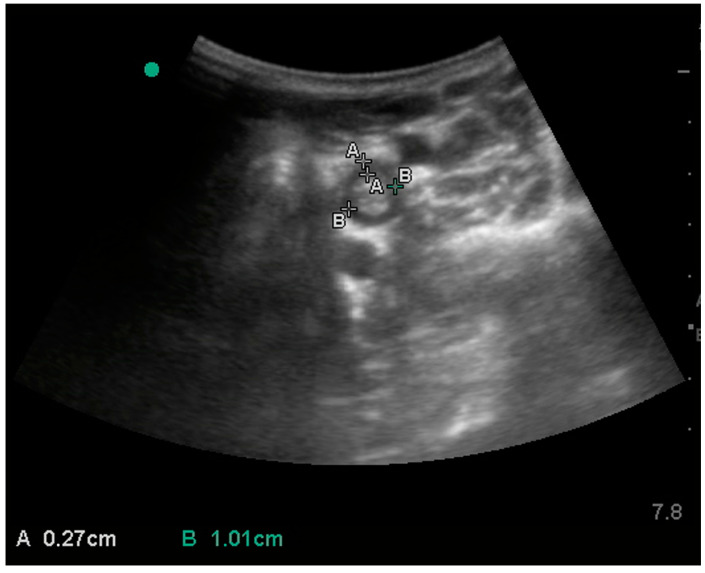
Ultrasonographic image of the oesophagus, obtained on the left upper neck with measurements of wall thickness (**A**) and diameter (**B**). The green dot represents the marker of the probe; the left side of this image is dorsal, and the right side is ventral.

For oesophagus evaluation, the acoustic window was located on the left side, caudally to the pharynx and ventrally in the proximal third of the neck (Figure 1); it was easily identified for the peristaltic waves, induced by administering a small amount of food (Figure 3).

The stomach wall was found to be visible in the left side, between the 9th and 13th ICS at the level of the scapulohumeral joint line. The visualization of the anechoic gastrosplenic vein was used as a marker, since the stomach wall is located immediately beyond this structure.

**Figure 4:**
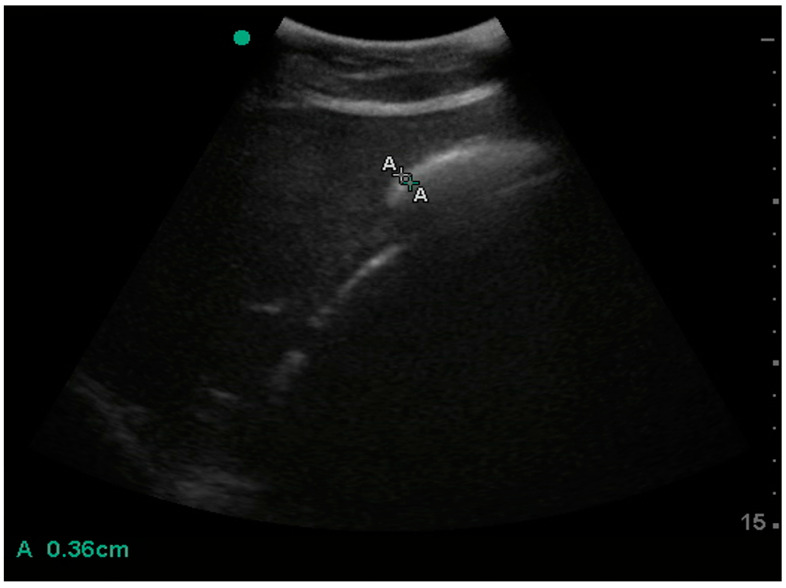
Ultrasonographic image of the duodenum with wall thickness measurement (A). The right dorsal colon is medial and the right liver lobe is lateral to the duodenum. The green dot represents the marker of the probe; the left side of this image is dorsal and the right side is ventral.

The duodenum was imaged in the right 15th ICS in all donkeys (Figure 4).

On the right side of the abdomen, the caecum was detected in the paralumbar fossa for 14 donkeys (82%), in the 17th ICS for 2 donkeys (12%) and in the 16th for 1 donkey (6%).

The left and right colon, both dorsal and ventral, were imaged in the ventral part of the abdomen on the left and right side, respectively, and were easily recognized for the absence of sacculation in the dorsal and for its presence in the ventral.

Regarding statistical evaluation, all data were found to be normally distributed. A significant correlation was found between the kidney length and the weight of the donkey.

## 4. Discussion

To the best of the authors’ knowledge, this is the first study reporting a complete ultrasonographic examination of the abdominal cavity in donkeys.

A comprehensive understanding of the normal ultrasonographic anatomy is essential, since US can be used for early diagnosis and colic recognition before the conditions become irreversible or life-threatening [17], since donkeys are stoic animals, and they are unlikely to show the dramatic signs of pain and distress that are exhibited by horses [9,12,18,19], even though they may be experiencing the same degree of pain [20].

The ultrasonographic examination of the abdomen was found to be safely and easily performed in donkeys under field conditions and without sedation. This study was conducted in the spring, and there was no need to clip the animals, even though some still had their winter coat; in fact, an adequate preparation of the hair using 90° alcohol was enough to achieve a good image quality.

The donkeys in this study were found to have more caudal and smaller kidneys compared to horses [21], as was expected due to the smaller size of the animal. However, our findings are similar to the ones reported by Hussein et al. about donkeys in terms of the length and thickness of the cortex, while the medulla had a significantly lower value compared to the present study. As previously described for both the donkey [22] and the horse [23], the animals in this study had kidneys of slightly different sizes, with the right being bigger than the left one.

Having reference values for the wall thickness of the gastrointestinal (GI) tract is crucial for comparisons with pathological conditions, since several diseases can cause infiltrative/inflammatory diseases with modification of the intestinal wall [3,24,25,26]. In 2020, Ibrahim and El-Ashker [8] established for the first time the position and wall thickness of different gastrointestinal portions in clinically healthy donkeys.

The recorded mean and standard deviations for the wall thickness of the stomach in the examined donkeys, visualized between the 9th and 13th ICS, were inside the range reported for donkeys [8] and for horses [27]. In all examined donkeys, the duodenum was constantly imaged at the right side of the abdomen in the 15th ICS, and the wall thickness was in line with the one previously described by Ibrahim and El-Ashker [8] for donkeys and Lester [21] and Bithell et al. [28] for horses. The large colon wall, both dorsal and ventral, was thicker than that of the horse [27,28], but thinner than the one reported by Ibrahim and El-Ashker in donkeys [8]. In the present study, the cecum was displayed between the 16th ICS and the paralumbar fossa, and the wall thickness was lower than the one described in the horse [28] and in the donkey [8].

The location and wall thickness measurement of the jejunum were not recorded, because they were not easily visualized in the dorsal and ventral abdomen on both sides, probably due to the reduced length of the viscera compared to the horse. This finding has also been reported by other authors [29].

Data regarding the comparison between the GI wall thickness and renal measurements of the present study with the available data on both horses and donkeys in the literature are summarized in Table 3.

This is in line with previous reports, since all measurements regarding the wall thickness of the gastrointestinal tracts were not correlated with age, nor with body weight [8].

One of the aims of this study was to determine the acoustic windows where the abdominal viscera could be found under field conditions. The findings of the present study are in some instances compatible with those reported in horses [21] for the spleen, stomach, duodenum, colon and cecum. However, the liver extension on the right side was found to shift more caudally than that in horses (horse: 5th to 15th ICS; donkey: 11th to 15th ICS), and the acoustic window was found to be three ICSs smaller [21]. This result is compatible with the ones previously reported by Hussein et al. [22] (donkey: 12th to 15th ICS).

The first limitation of this study lies in the fact that there is not enough research on donkeys to compare the results we obtained with the literature. Comparing the measurements of standard donkeys with those of horses could create a bias related to the size of the animals, even if Epstein et al. [5] reported wall thickness measurements of different GI viscera in 108–216 kg ponies compared to horses, and the findings of the present study are, again, more in line with the ones reported for horses [32,33,34]. In any case, further studies could confirm whether there is a correlation between measurements and the size of donkeys being more variable in terms of weight than it was in our population. Another limitation of this study is that the animal population includes only females. Further studies could highlight possible gender differences regarding the anatomical location and measurement of abdominal viscera compared to males.

## 5. Conclusions

In conclusion, this study defined, for the first time, reference values for the probe position to be used for abdominal scanning in the field in healthy jennies. We also reported the wall thicknesses of the different gastrointestinal viscera in clinically healthy donkeys, which were found to only be partially related to the ones of the horse. In the present study, no pathological condition was investigated. In any case, it can provide a collection of basic data for further use for ultrasonographic scanning of a donkey’s abdomen as an important diagnostic tool to achieve an early diagnosis of pathological changes. Due to the low tendency of donkeys to show clinical symptoms of pain or discomfort compared to horses, ultrasound acquires considerable importance as an early diagnostic tool in the evaluation of this species.

## Figures and Tables

**Table 1:** Descriptive statistics of the measurements.

Variable	Measure (mm)	Mean (SD)	95% IC	Percentile				Min	Max	Range
				10%	25%	75%	90%			
Left kidney	length	91.5 (9.0)	88.0–96.8	84.6	88.7	96.5	99.0	66.3	108.0	41.7
	cortex	8.4 (2.2)	6.7–8.9	6.3	6.9	8.9	10.9	5.3	13.9	8.6
	medulla	15.6 (5.5)	11.6–17.0	10.1	11.4	18.8	22.5	8.0	27.4	19.4
Right kidney	length	95.5 (10.8)	88.1–98.7	82.6	89.8	102.3	110.0	77.5	113.0	35.5
	cortex	8.4 (1.6)	7.7–9.3	6.4	7.5	9.6	10.4	5.8	11.4	5.6
	medulla	15.3 (2.0)	14.1–16.1	13.1	14.8	16.8	17.2	10.5	18.8	8.3
Oesophagus	wall thickness	3.6 (0.7)	3.5–4.1	2.7	3.0	4.1	4.3	2.3	4.9	2.6
	diameter	18.9 (4.8)	18.5–23.3	11.4	14.4	22.3	23.5	10.1	24.6	14.5
Stomach	wall thickness	6.6 (1.1)	5.5–6.7	5.6	5.7	7.0	8.2	5.2	9.1	3.9
Duodenum	wall thickness	3.5 (0.6)	3.1–3.7	3.0	3.3	3.8	4.0	2.2	5.3	3.1
Cecum	wall thickness	3.5 (0.9)	3.2–4.0	2.5	3.0	4.1	4.3	1.4	4.8	3.4
Left colon	wall thickness	4.4 (1.0)	3.8–4.8	3.4	3.8	5.3	5.7	2.6	6.4	3.8
Right colon	wall thickness	4.2 (0.9)	3.4–4.4	2.8	3.2	4.4	5.3	2.4	5.5	3.1

**Table 2:** Association between measurements (mm) and age and body weight. r = correlation coefficient. Asterisk indicates significant correlation.

Variable	Measure (mm)	Age		Weight	
		r	*p*-Value	r	*p*-Value
Left kidney	length	0.401	0.062	0.587	0.008 *
	cortex	−0.039	0.443	0.323	0.047
	medulla	0.138	0.305	0.433	0.047
Right kidney	length	0.301	0.128	0.669	0.002 *
	cortex	−0.054	0.421	0.155	0.284
	medulla	0.054	0.422	0.275	0.151
Oesophagus	wall thickness	−0.131	0.685	0.465	0.035
	diameter	0.267	0.159	0.241	0.184
Stomach	wall thickness	0.394	0.065	−0.384	0.071
Duodenum	wall thickness	−0.052	0.425	0.317	0.116
Cecum	wall thickness	−0.401	0.062	−0.368	0.080
LC	wall thickness	0.020	0.471	0.422	0.052
RC	wall thickness	−0.418	0.053	0.390	0.068

**Table 3:** Comparison of measurements between the present study and the literature for horses and donkeys.

		**Donkey** **(Present Study)**	**Donkey (The Literature)**	**Horse (The Literature)**
**Variable**	**Measure (mm)**	**Mean (SD)**	**Mean (SD)**	**Mean (SD)**
Left kidney	length	91.5 (9.0)	89 (9.0) [1]	151 (17) [3,4]
	cortex	8.4 (2.2)	8.3 (9.0) [1]	11 (2) [3]
	medulla	15.6 (5.5)	8.4 (7.0) [1]	23 (3) [3]
Right kidney	length	95.5 (10.8)	100 (8.0) [1]	168 (12) [3,4]
	cortex	8.4 (1.6)	8.6 (8.0) [30]	12 (2) [31]
	medulla	15.3 (2.0)	8.5 (9.0) [30]	28 (3) [31]
Oesophagus	wall thickness	3.6 (0.7)		
	diameter	18.9 (4.8)		
Stomach	wall thickness	6.6 (1.1)	7.0 (0.9) [8]	<7 [21,27]
Duodenum	wall thickness	3.5 (0.6)	3.3 (1.0) [8]	3 (0.4) [21,28]
Cecum	wall thickness	3.5 (0.9)	5.4 (0.6) [8]	4.2 (0.3) [21,28]
Left colon	wall thickness	4.4 (1.0)	5.1 (0.5) [8]	3.7 (0.1) [21,27,28]
Right colon	wall thickness	4.2 (0.9)	5.4 (0.6) [8]	3.7 (0.1) [21,27]

## Data Availability

The original contributions presented in this study are included in the article. Further inquiries can be directed to the corresponding author.
